# Supplementary material for: Gastruloids are competent to specify both cardiac and skeletal muscle lineages
Source: Nat Commun. 2024 Nov 23;15:10172. doi: 10.1038/s41467-024-54466-w (PMC11585638; doi:10.1038/s41467-024-54466-w)
Supplement: Supplementary file 1 — Supplementary Information [file 41467_2024_54466_MOESM1_ESM.pdf]

Supplementary information

Gastruloids are competent to specify both cardiac and skeletal muscle lineages

Laurent Argiro [1, $], Céline Chevalier [1, $], Caroline Choquet [1, $], Nitya Nandkishore [1, 2], Adeline Ghata [1], Anaïs Baudot [1], Stéphane Zaffran [1, £]*, Fabienne Lescroart [1, £]*

[1] Aix-Marseille Univ, INSERM, Marseille Medical Genetics (MMG), Marseille, France.

[2] Present address: Department of Biotechnology, SRM Institute of Science and Technology (SRMIST), Kattankulathur - 603 203, Tamil Nadu, India.

[$] These authors contributed equally

[£] These authors jointly supervised this work

* Corresponding authors: fabienne.lescroart@univ-amu.fr, stephane.zaffran@univ-amu.fr

Supplementary Table 1: Differential gene expression analysis (between the cardiomyocytes clusters of day 11)

Differentially expressed genes (DEGs) were determined using a Wilcoxon Rank Sum test with the Seurat function FindAllMarkers (one-sided test).

cluster	gene	avg_log2FC	pct.1	pct.2	p_val	p_val_adj
6	Actg1	1.9520497598933	1	0.98	9.3292424993531E-145	1.86053083164599E-140
6	Actb	2.13442707317024	0.992	0.933	1.47819468257727E-121	2.94796365546385E-117
6	Acta2	2.68484766092077	0.942	0.608	6.15351234694026E-117	1.2271949673503E-112
6	Cald1	1.53253259486526	0.934	0.62	2.23908523204642E-115	4.46540767827017E-111
6	Vsnl1	1.52489294166401	0.811	0.334	7.30934997371835E-97	1.45770366525865E-92
6	Gucy1a1	1.29918997463378	0.886	0.637	2.80083956814816E-80	5.58571435075788E-76
6	Tpm2	1.257035819452	0.916	0.701	4.59570578360096E-74	9.16521604423539E-70
6	Shox2	1.19964209190323	0.843	0.415	1.98337277675618E-66	3.95544032868485E-62
6	Ptn	2.23634395737377	0.811	0.513	2.86394482682357E-66	5.71156516813425E-62
6	Maged2	1.18372950759593	0.962	0.851	6.3454307639539E-62	1.26546925725533E-57
6	Meg3	1.50051497903603	0.871	0.595	9.24182981137988E-62	1.84309811928349E-57
6	Mdk	1.10488062248775	0.95	0.805	4.01236480067597E-55	8.00185912198808E-51
6	Cdkn1c	1.57559149761615	0.958	0.906	4.07081202660618E-55	8.11842042466071E-51
6	Prnp	1.07638223833597	0.861	0.756	5.77542993384708E-50	1.15179399170712E-45
6	Tagln	1.79456404971038	0.357	0.071	1.70746117533773E-45	3.40518982197603E-41
6	Pnmt	1.36580058833633	0.536	0.225	4.11175508636781E-39	8.20007316874332E-35
6	Csrp2	1.25999558667678	0.97	0.964	4.18961116963935E-29	8.35534155561176E-25
6	Mgp	1.11264458561913	0.516	0.308	3.78991869328861E-24	7.55823485002547E-20
6	Gal	1.32615716611783	0.219	0.105	0.000000000758751728	0.00001513178571312
6	Fabp5	1.26011794397178	0.882	0.853	0.000000011982820195	0.000238973383146723
8	Myl2	3.91924440186805	0.952	0.346	5.27177377234951E-167	1.05134984341966E-162
8	Myl3	2.87809664062242	0.984	0.559	4.41193166838023E-155	8.79871532625069E-151
8	Myh7	3.01672448698971	0.982	0.669	1.28109789427715E-145	2.55489353055692E-141
8	Mpped2	1.34135916286582	0.84	0.197	2.77773947114668E-134	5.53964582730782E-130
8	Pln	2.13067249544387	0.936	0.494	3.59481088311273E-112	7.16913134419172E-108
8	Hopx	1.18782692538119	0.884	0.427	9.23373034467432E-88	1.8414828426384E-83
8	Retreg1	0.603194149438958	0.573	0.095	2.72638502325051E-87	5.43722965186849E-83
8	Mhrt	0.724323835327955	0.557	0.107	1.91874214902498E-76	3.82654746780053E-72
8	Spink4	1.27694594691667	0.671	0.24	4.4274041281581E-67	8.82957205278569E-63
8	Mb	0.939573463172259	0.605	0.234	4.19177329513539E-54	8.3596534824885E-50
8	Crip2	0.701507105371309	0.991	0.941	5.40765839945553E-51	1.07844931460342E-46
8	Actn2	0.708032558494068	0.943	0.699	1.02100697320104E-46	2.03619420665484E-42
8	Fhl2	0.959723969666818	0.842	0.636	8.2634084299767E-46	1.64797154319025E-41
8	Mllt3	0.717005407346828	0.776	0.485	7.76876246428685E-43	1.54932429825273E-38
8	Cyba	0.604797659948406	0.9	0.712	1.37263368048981E-41	2.73744334900083E-37
8	Gyg	0.619106891466274	0.998	0.987	3.83198467925149E-41	7.64212704583124E-37
8	Kcne1	0.648115701807294	0.5	0.181	7.29894536982245E-39	1.45562867510369E-34
8	Cox8b	0.614498061937409	0.79	0.508	3.59412544647057E-33	7.16776437789626E-29
8	2410006H16Rik	0.688849147010127	0.973	0.894	6.00894579360899E-33	1.19836405961944E-28
8	Cited1	0.969814821272424	0.662	0.418	6.17370735109916E-25	1.23122245702971E-20
9	Myh6	1.59235974577339	1	0.862	3.97446437709652E-105	7.92627430724359E-101
9	Casq1	1.79759134202071	0.883	0.494	1.91745079450269E-86	3.82397211947671E-82
9	Myl7	0.817380821068884	1	1	1.32413621983951E-67	2.64072486322594E-63
9	Pam	1.0724249042968	0.984	0.876	1.22914310728383E-66	2.45128009885614E-62
9	Myl4	0.757030806112411	1	0.997	1.73885433631051E-65	3.46779720290405E-61
9	Itga6	1.09158197561414	0.81	0.44	2.25547832085908E-65	4.49810041528927E-61
9	Tesc	0.972035422900798	0.892	0.623	1.7106469861392E-56	3.41154328445741E-52
9	Stard10	0.885684559827296	0.874	0.564	5.29535366765539E-55	1.05605238194051E-50
9	Palld	0.912296477734801	0.963	0.828	2.76924818275449E-54	5.52271165086727E-50
9	Slc8a1	0.851869941194078	0.991	0.927	7.73181438459535E-52	1.54195574271985E-47
9	Smpx	0.808396923233642	0.953	0.767	8.92677490500744E-47	1.78026671930563E-42
9	Ccnd2	0.877567370057972	0.946	0.738	2.75286870172281E-46	5.49004605184581E-42
9	Obscn	0.824131869765032	0.93	0.694	5.16813613304334E-46	1.03068138901283E-41
9	Mlf1	0.833262290040485	0.892	0.7	2.20424632681714E-42	4.39592844957143E-38
9	Sln	1.60475410018664	0.445	0.131	1.07163298871321E-40	2.13715766939075E-36
9	Atcayos	0.739159367184943	0.977	0.81	1.95143439768714E-36	3.89174561930747E-32
9	Calca	0.902898889852385	0.614	0.254	3.74568819625215E-36	7.47002596978566E-32
9	Nppa	1.94085644980968	0.319	0.079	1.73789152270502E-31	3.46587706373062E-27
9	Ankrd1	0.904934716717817	0.543	0.262	7.29936675209728E-26	1.45571271137076E-21
9	Mest	1.17185018930633	0.686	0.481	1.43322925683164E-23	0.0000000000000000002

Supplementary Table 2: qPCR primers

gene	forward	reverse	product length
Isl1	GCATCATGATGAAGCAGCTC	CATCGATGCTACTTCACTGC	275
Mesp1	GTCTGCAGCGGGGTGTCGTG	CGGCGGCGTCCAGGTTTCTA	189
Myl2	CCCAGATCCAGGAGTTCAAG	CTGGTCGATCTCCTCTTTGG	341
Myl7	ATCCTGAGTGCCTTCCGCATG	GGTGTCAGCGCAAACACTTGC	134
TBP	CCCCACAACTCTTCCATTCT	GCAGGAGTGATAGGGGTCAT	103
Tbx1	CGAGATGATCGTCACCAAGG	CCAGGAGGAGCTATGGAAAG	154
Tcf21	CTGTAGTTCCACACAAGCGG	CGGTTACATTCACCCAGTCA	107
Myf5	GACAGGGCTGTTACATTCAGG	TGAGGGAACAGGTGGAGAAC	110
Tnnt2	CAAGGAGCTGTGGCAGAGTA	TTCTGGTTGTCATTGATCCG	120
MyoD	GTCGTAGCCATTCTGCCG	AGCACTACAGTGGCGACTCA	110
Ebf3	AGAGCCGAACAACGAGAAAA	GCACATCTCCGGATTCTTGT	163

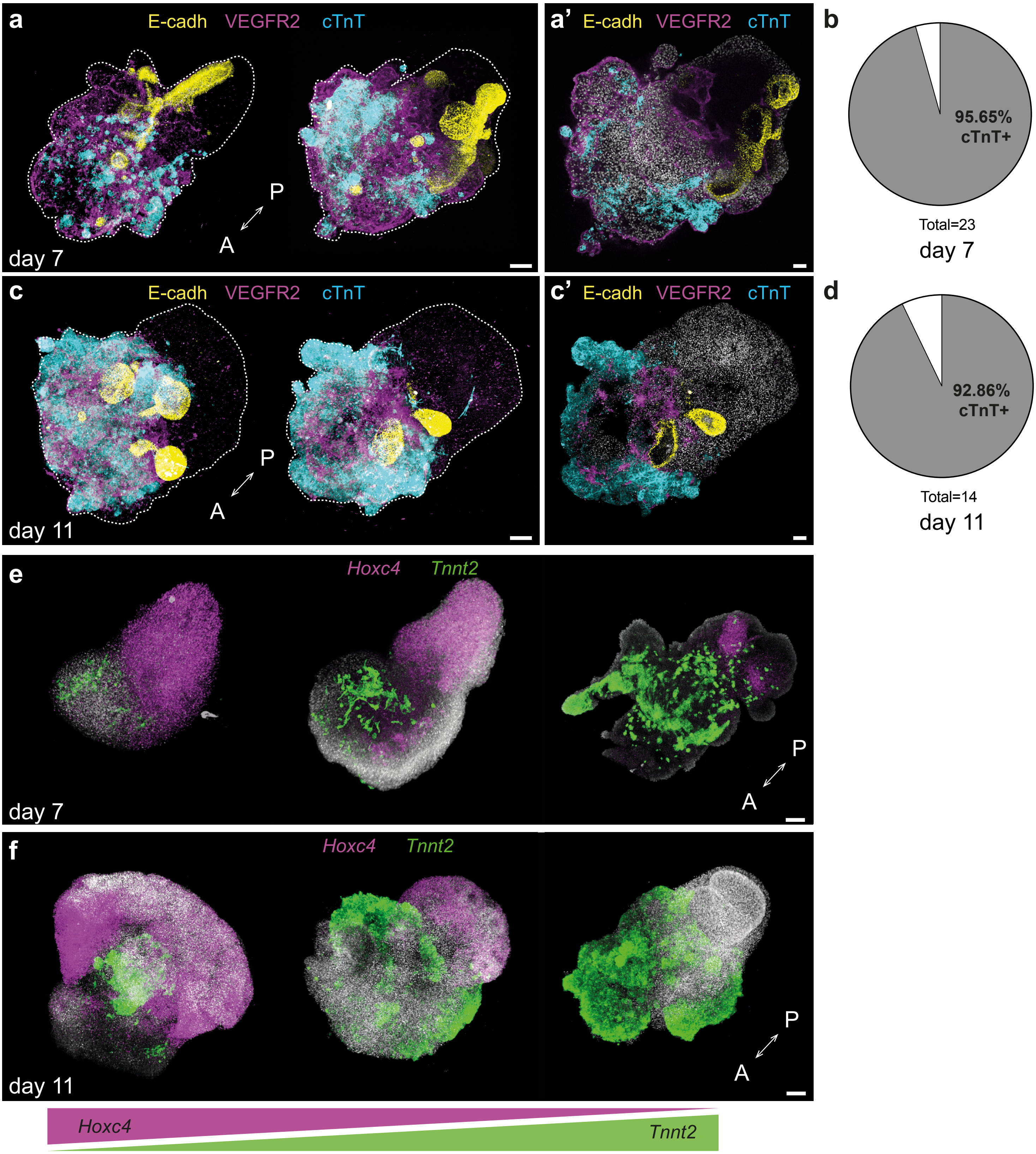

Supplementary Figure 1. Description of the morphology of day 7 and day 11 gastruloids.
a. Representative maximum intensity projection images of two gastruloids on day 7 after immuno-fluorescence with E-cadherin (yellow), VEGFR2 (purple) and cTNT (cyan) antibodies. An optical section of the z-stack is shown in **a'**. **b.** Percentage of gastruloids on day 7 with cTnT expression (n=23 in n=5 independent experiments). **c.** Representative maximum intensity projection images of two gastruloids on day 11 after immune-fluorescence with E-cadherin (yellow), VEGFR2 (purple) and cTNT (cyan) antibodies). An optical section of the z-stack is shown in **c'**. **d.** Percentage of gastruloids on day 11 with cTnT expression (n=14 in n=5 independent experiments). **e-f.** Representative maximum intensity projection images of gastruloids on day 7 (e)and on day 11 (f) after RNAscope with *Tnnt2* (green) and *Hoxc4* (purple) probes. Scales: 100μm. A, anterior, P, posterior.

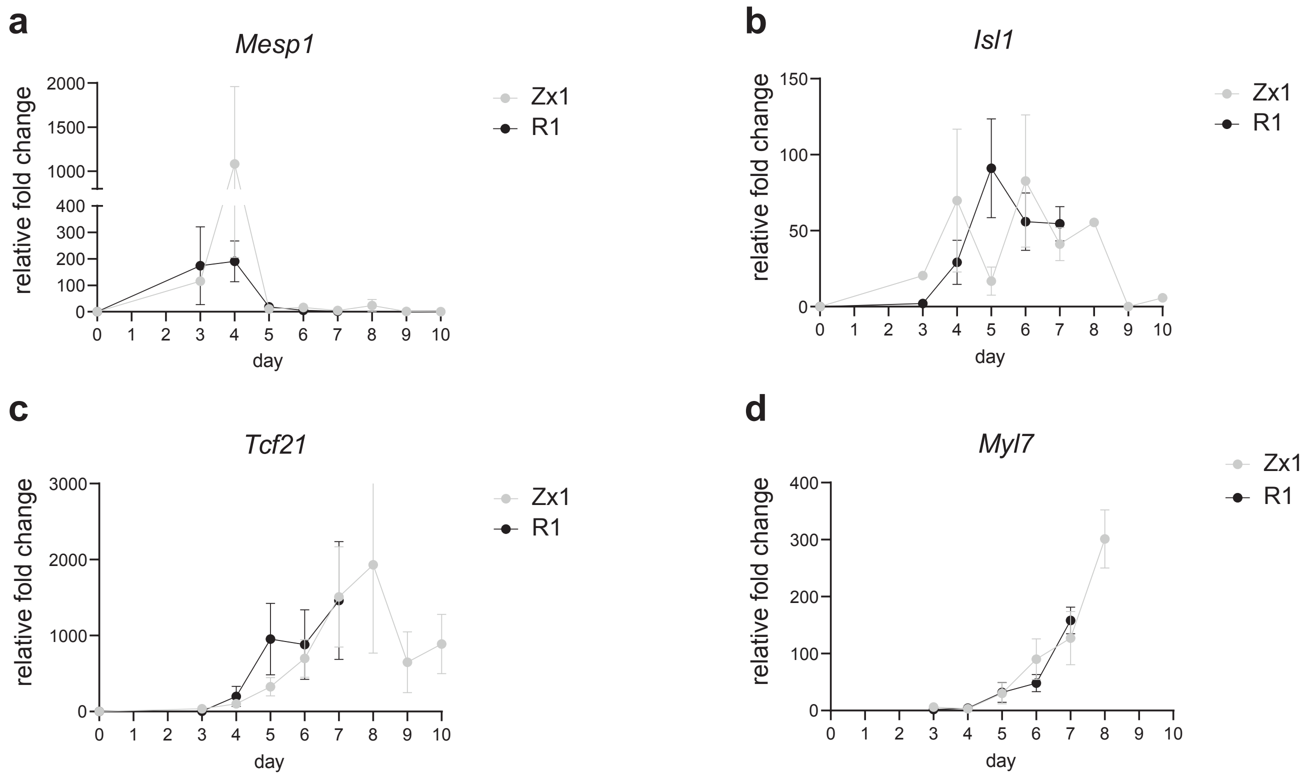

Supplementary Figure 2. Similar expression kinetics with 2 different cell lines.
Expression profiles of *Mesp1* (Zx1-n=7; R1-n=5) (**a**), *Isl1* (Zx1-n=3; R1-n=5) (**b**), *Tcf21* (Zx1-n=7; R1-n=5) (**c**) and *Myl7* (Zx1-n=5; R1-n=4) (**d**) throughout the culture of gastruloids with Zx1 (grey) and R1 (black) cell lines as measured by quantitative RT-PCR. Results are normalized on the expression of *TBP*. Fold changes are represented over expression day 0. (Mean with standard error of mean (SEM) (n>3).

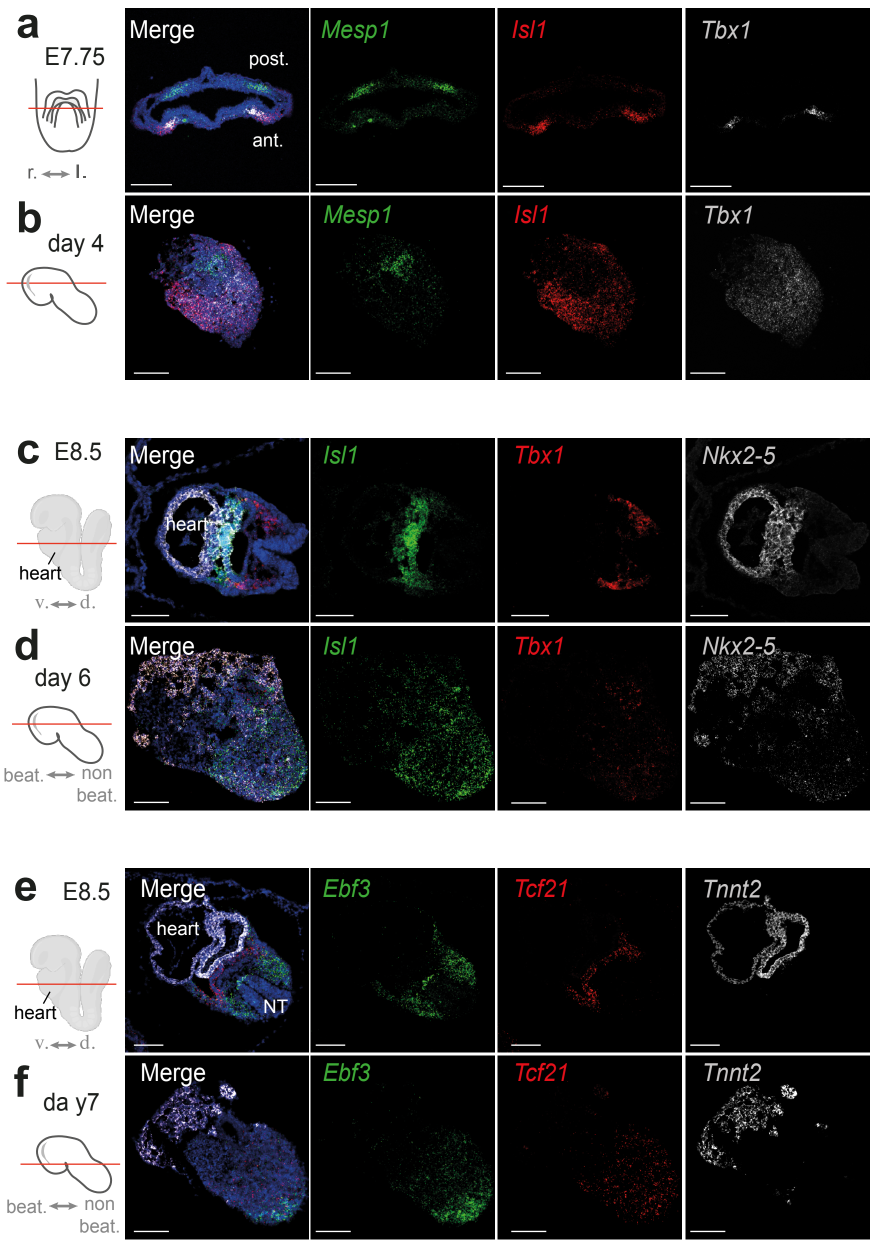

Supplementary Figure 3. Comparison of expression patterns between embryos and gastruloids.
a-b. Representative confocal images of sections across an E7.75 mouse embryo (**a**) and a gastruloid at late day 4 (**b**) after RNAscope experiment with *Mesp1* (green), *Isl1* (red) and *Tbx1* (white) probes. **c-d.** Representative confocal images of sections across an E8.5 mouse embryo (**c**) and a gastruloid at day 6 (**d**) after RNAscope experiment with *Isl1* (green), *Tbx1* (red) and *Nkx2-5* (white) probes. **e-f.** Representative confocal images of sections across an E8.5 mouse embryo (**e**) and a gastruloid at day 7 (**f**) after RNAscope experiment with *Ebf3* (green), *Tcf21* (red) and *Tnnt2* (white) probes. r, right; l, left. v, ventral; d, dorsal; beat., beating; non beat., non-beating., ant., anterior, post., posterior, NT, neural tube. Scale bars: 100 μm.

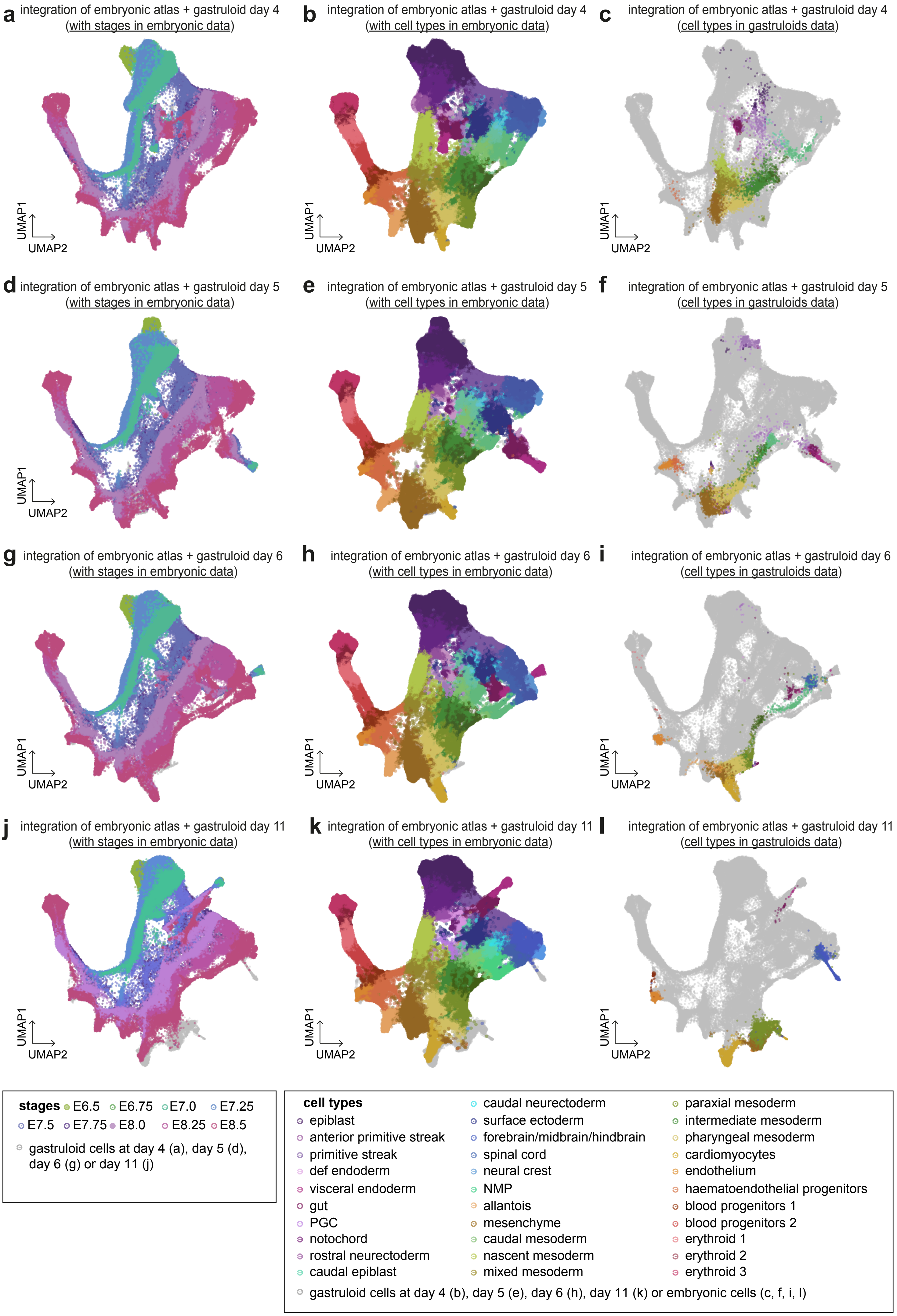
a integration of embryonic atlas + gastruloid day 4 (with stages in embryonic data)
b integration of embryonic atlas + gastruloid day 4 (with cell types in embryonic data)
c integration of embryonic atlas + gastruloid day 4 (cell types in gastruloids data)
d integration of embryonic atlas + gastruloid day 5 (with stages in embryonic data)
e integration of embryonic atlas + gastruloid day 5 (with cell types in embryonic data)
f integration of embryonic atlas + gastruloid day 5 (cell types in gastruloids data)
g integration of embryonic atlas + gastruloid day 6 (with stages in embryonic data)
h integration of embryonic atlas + gastruloid day 6 (with cell types in embryonic data)
i integration of embryonic atlas + gastruloid day 6 (cell types in gastruloids data)
j integration of embryonic atlas + gastruloid day 11 (with stages in embryonic data)
k integration of embryonic atlas + gastruloid day 11 (with cell types in embryonic data)
l integration of embryonic atlas + gastruloid day 11 (cell types in gastruloids data)
UMAP1
UMAP2
stages E6.5 E6.75 E7.0 E7.25 E7.5 E7.75 E8.0 E8.25 E8.5
gastruloid cells at day 4 (a), day 5 (d), day 6 (g) or day 11 (j)
cell types
epiblast
anterior primitive streak
primitive streak
def endoderm
visceral endoderm
gut
PGC
notochord
rostral neurectoderm
caudal epiblast
caudal neurectoderm
surface ectoderm
forebrain/midbrain/hindbrain
spinal cord
neural crest
NMP
allantois
mesenchyme
caudal mesoderm
nascent mesoderm
mixed mesoderm
paraxial mesoderm
intermediate mesoderm
pharyngeal mesoderm
cardiomyocytes
endothelium
haematoendothelial progenitors
blood progenitors 1
blood progenitors 2
erythroid 1
erythroid 2
erythroid 3
gastruloid cells at day 4 (b), day 5 (e), day 6 (h), day 11 (k) or embryonic cells (c, f, i, l)

Supplementary Figure 4. Analysis of single-cell gastruloids data integrated with the embryonic atlas. UMAP representations of the mouse embryonic cell atlas integrated with gastruloids' single cell datasets. The first line represents the embryonic atlas + day 4 (**a-c**). The second line represents the embryonic atlas + day 5 (**d-f**). The third line represents the embryonic atlas + day 6 (**g-i**) and the fourth line the embryonic atlas + day 11 (**j-l**). The first column (**a**, **d**, **g** and **j**) shows in color only embryonic cells (with colors indicating the stage of collection) and gastruloids cells are shown in grey. Legends are found below j. The second column (**b**, **e**, **h** and **k**) shows only embryonic cells (with colors indicating the cell identity) and gastruloids cells are shown in grey. Legends are found below k. The third column (**c**, **f**, **i** and **l**) shows only gastruloids cells at day 4 (c), day 5 (f), day 6 (i) or day 11 (l) (with colors indicating the cell label based on the embryo atlas). Cells in grey correspond to the embryo. Legends are found below l.

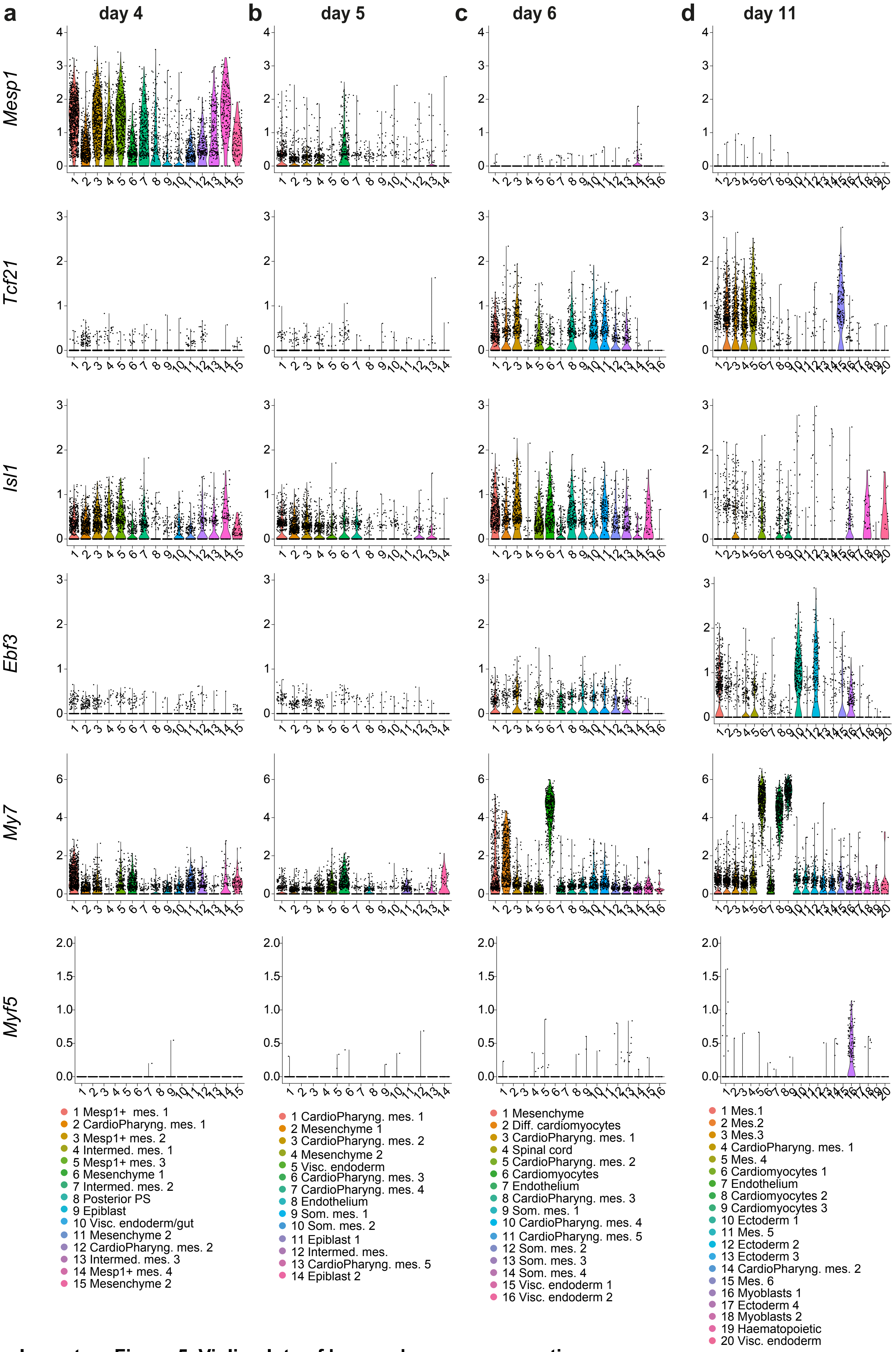

Supplementary Figure 5. Violin plots of key markers genes over time.
Violin Plots showing expression of *Mesp1*, *Tcf21*, *Isl1*, *Ebf3*, *Myl7* and *Myf5* at day 4 (**a**), day 5 (**b**), day 6 (**c**) and day 11 (**d**).

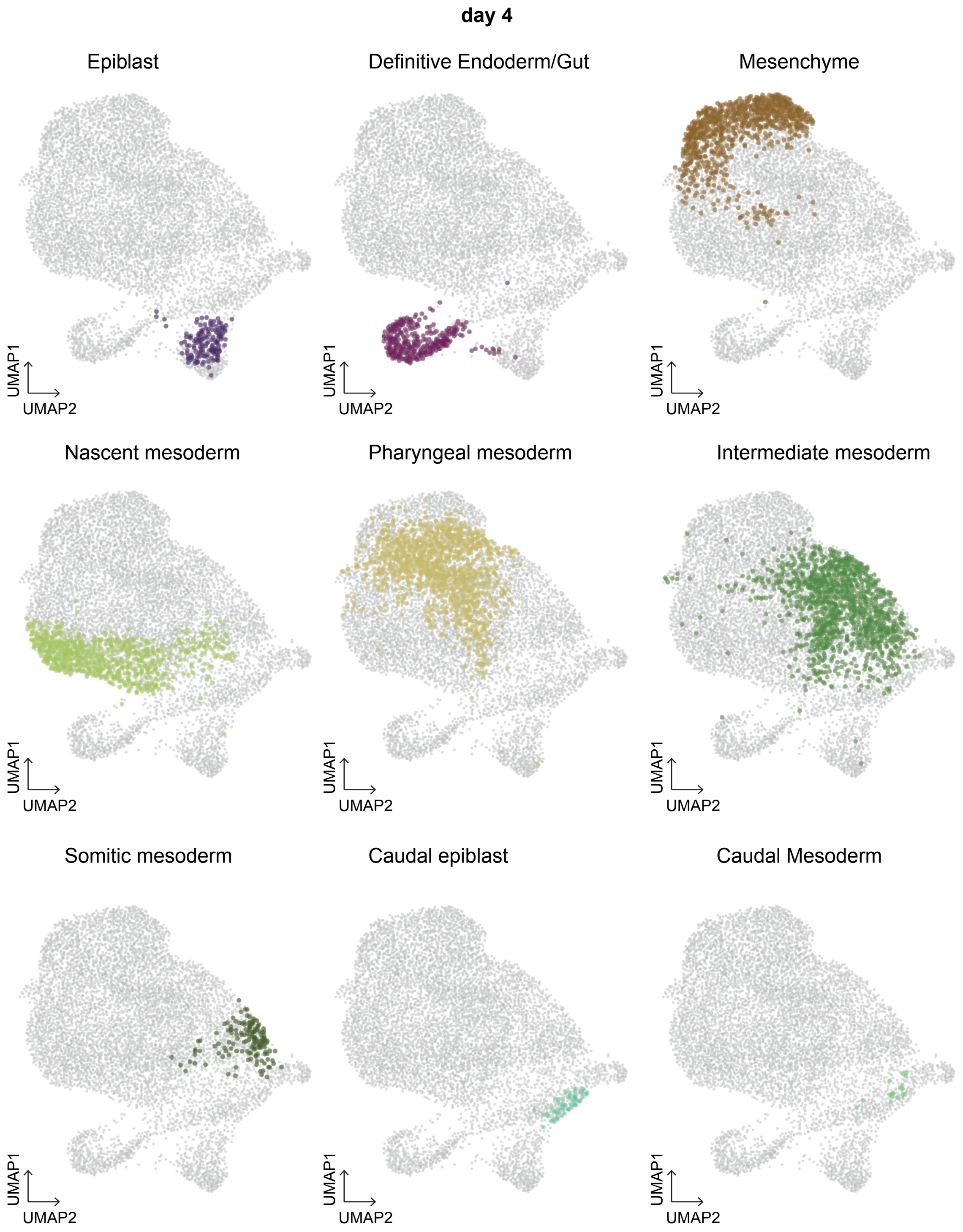

Supplementary Figure 6. Predictive cell types from the embryonic cell atlas in gastruloids at day 4. UMAP representations of gastruloids' single cell datasets at day 4 with predicted cell types from the embryonic cell atlas.

day 4

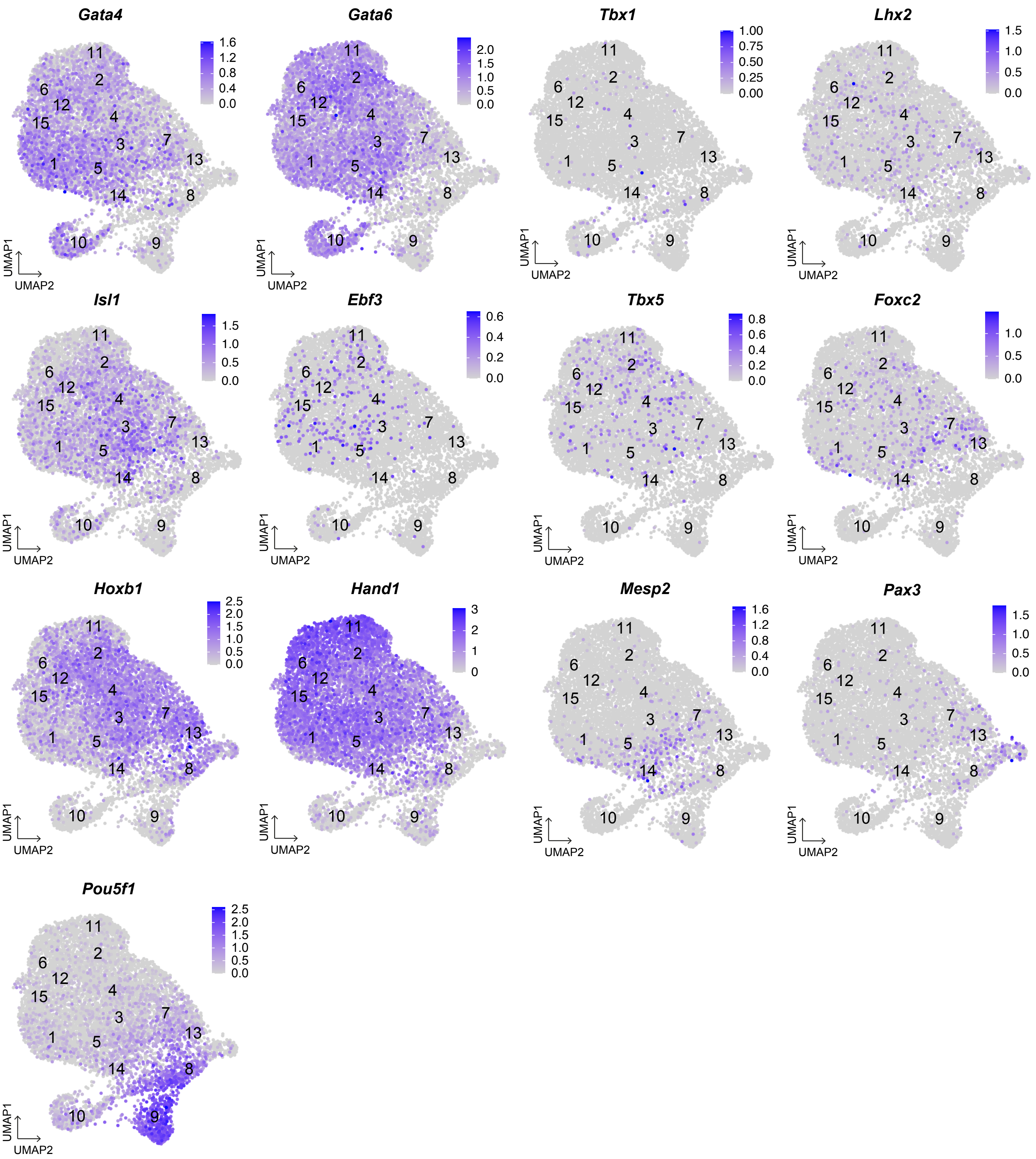

Supplementary Figure 7. Expression of key markers in single-cell RNAseq of gastruloids at day 4. Feature Plots showing expression of *Gata4*, *Gata6*, *Tbx1*, *Lhx2*, *Isl1*, *Ebf3*, *Tbx5*, *Foxc2*, *Hoxb1*, *Hand1*, *Mesp2*, *Pax3* and *Pou5f1* in gastruloids at day 4. Scale bars represent expression levels. Numbers represent Leiden clusters.

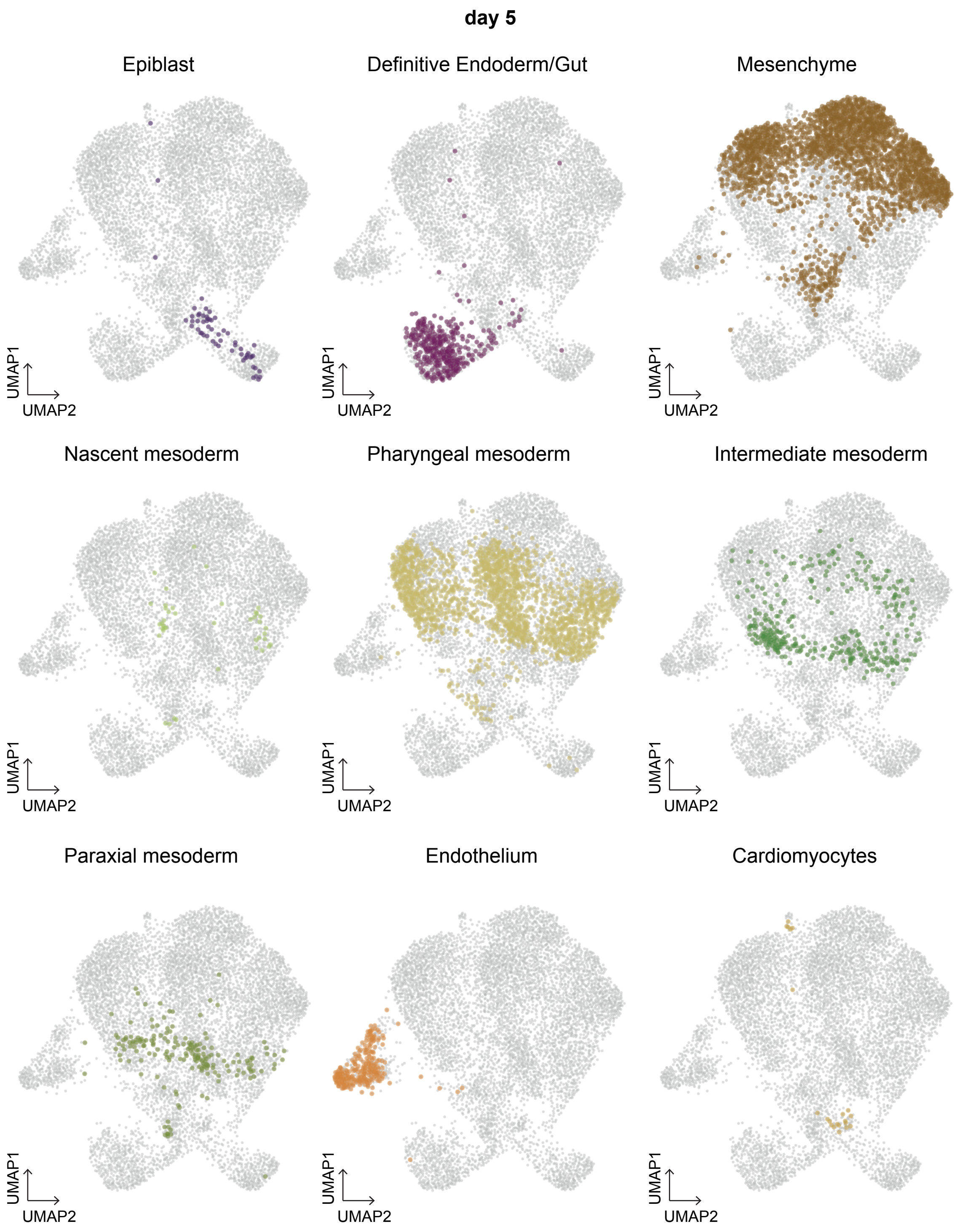

Supplementary Figure 8. Predictive cell types from the embryonic cell atlas in gastruloids at day 5. UMAP representations of gastruloids' single cell datasets at day 5 with predicted cell types from the embryonic cell atlas.

day 5

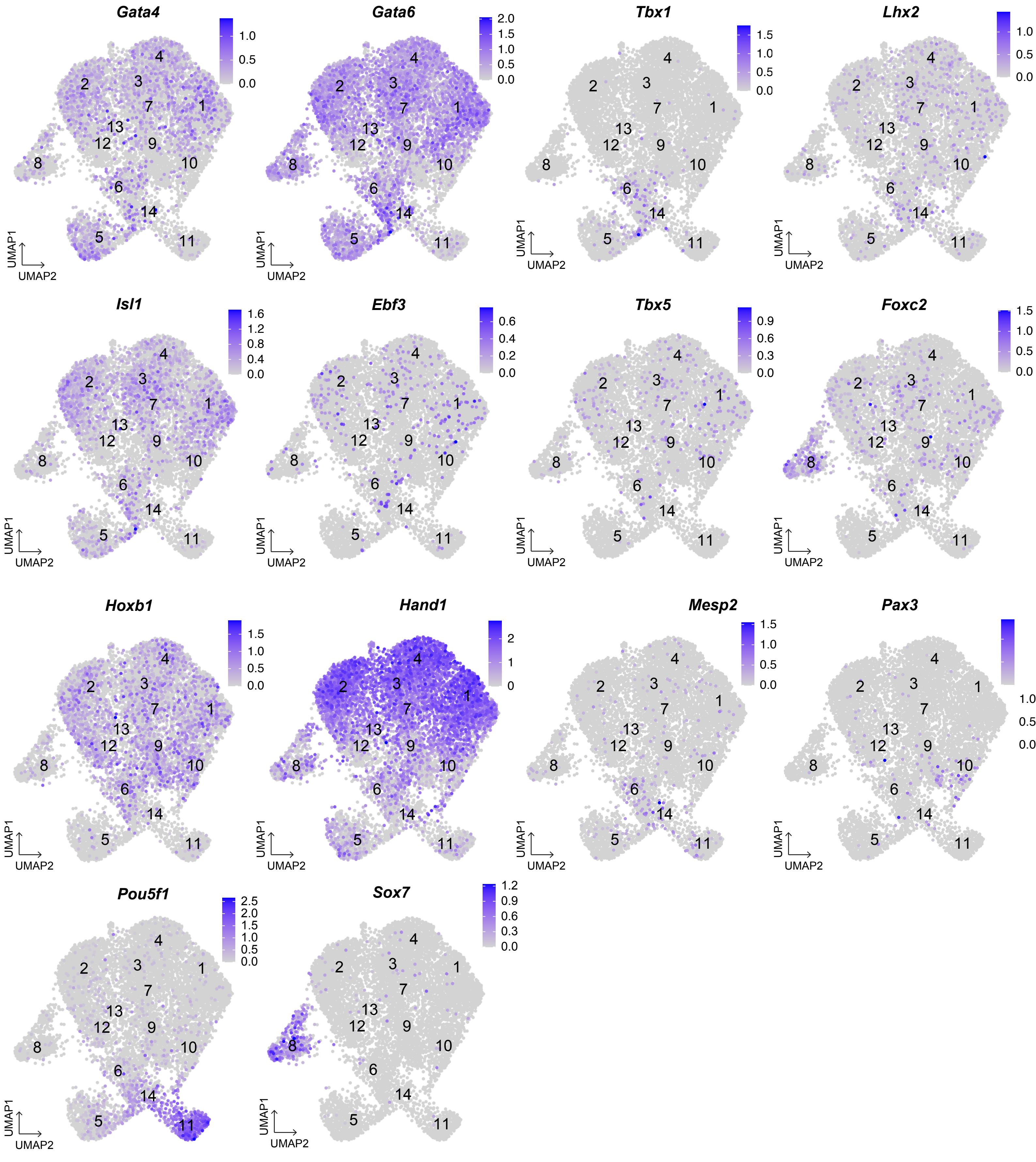

Supplementary Figure 9. Expression of key markers in single-cell RNAseq of gastruloids at day 5. Feature Plots showing expression of *Gata4*, *Gata6*, *Tbx1*, *Lhx2*, *Isl1*, *Ebf3*, *Tbx5*, *Foxc2*, *Hoxb1*, *Hand1*, *Mesp2*, *Pax3, Pou5f1* and *Sox7* in gastruloids at day 5. Scale bars represent expression levels. Numbers represent Leiden clusters.

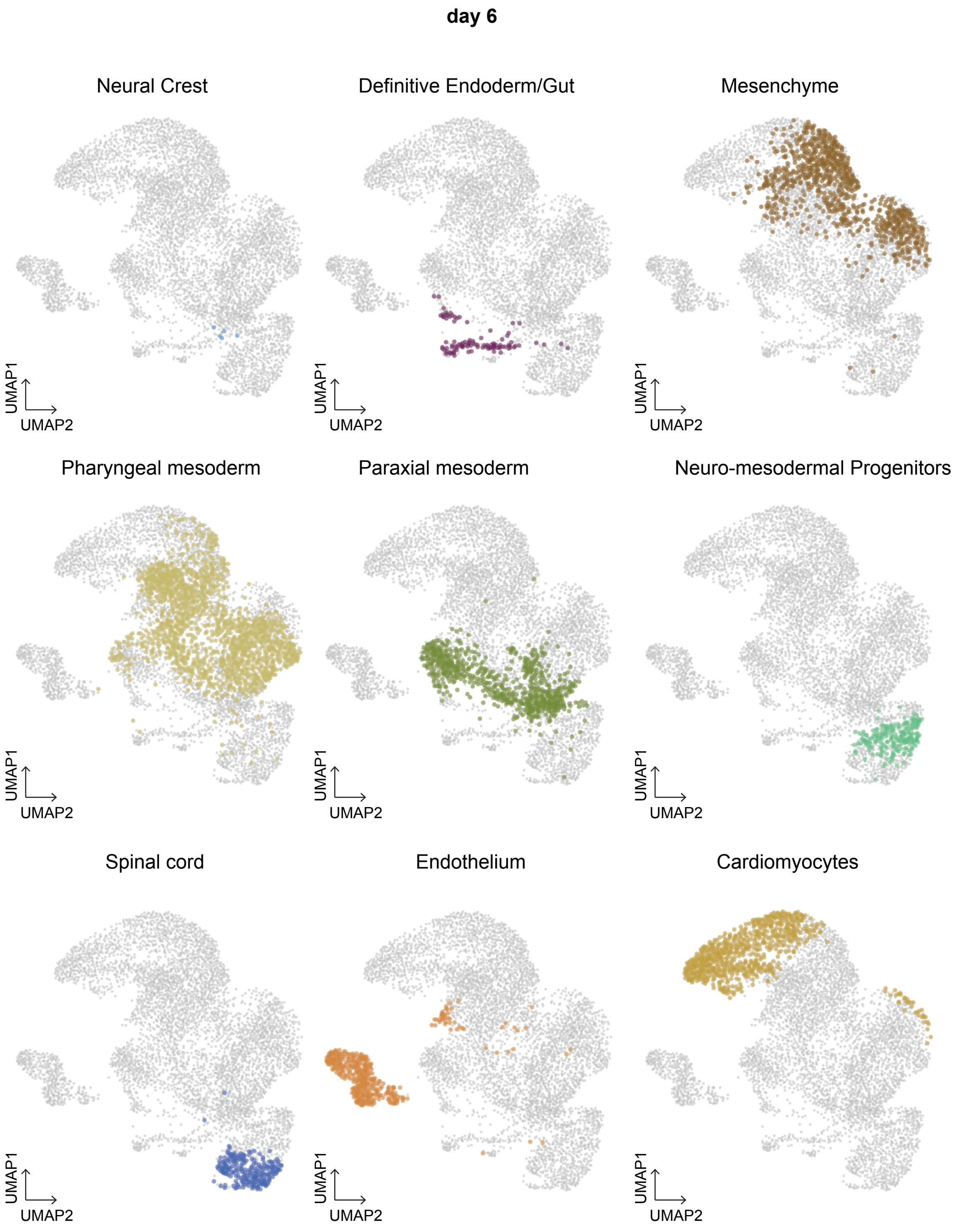

Supplementary Figure 10. Predictive cell types from the embryonic cell atlas in gastruloids at day 6. UMAP representations of gastruloids' single cell datasets at day 6 with predicted cell types from the embryonic cell atlas.

day 6

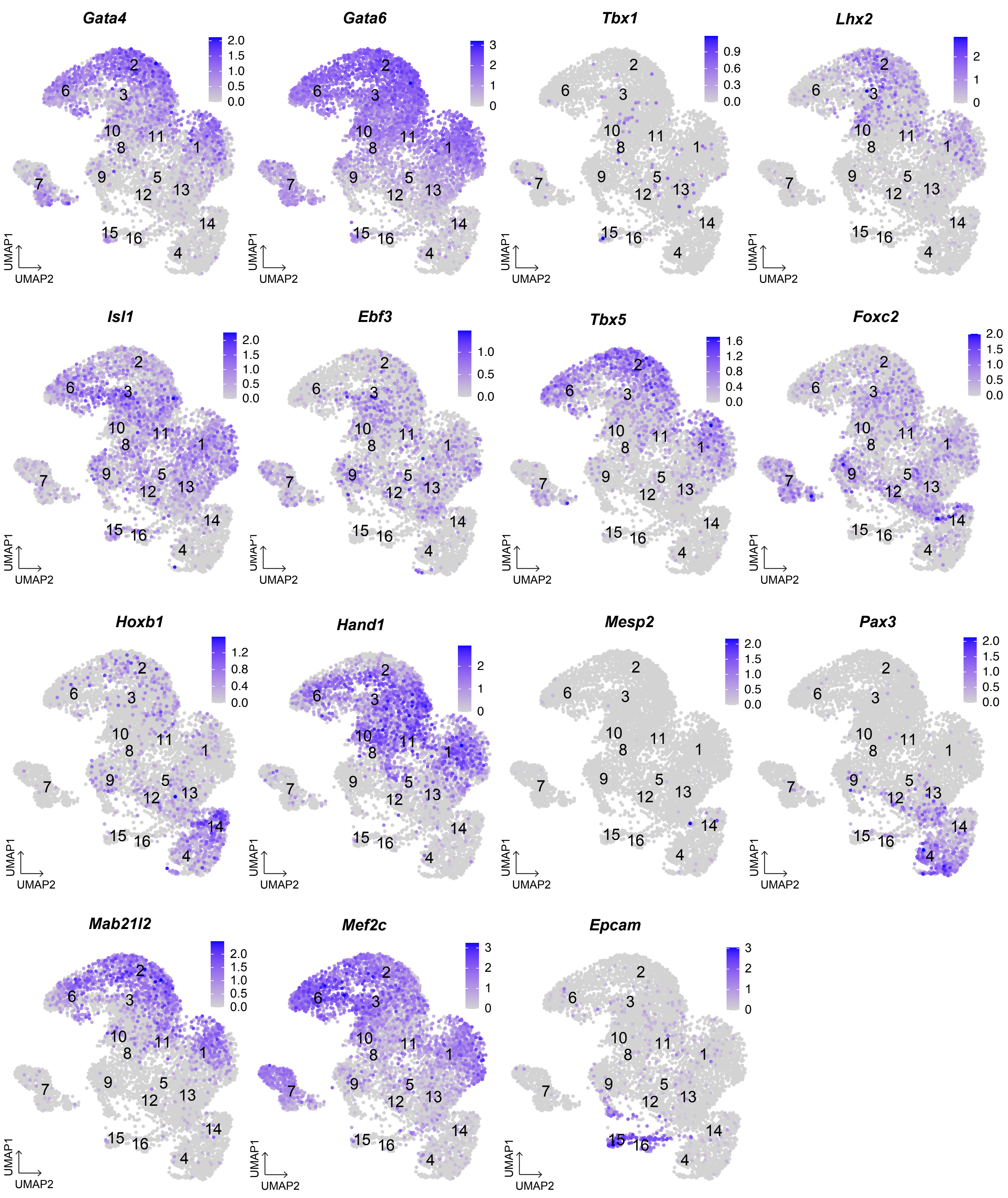

Supplementary Figure 11. Expression of key markers in single-cell RNAseq of gastruloids at day 6. Feature Plots showing expression of *Gata4*, *Gata6*, *Tbx1*, *Lhx2*, *Isl1*, *Ebf3*, *Tbx5*, *Foxc2*, *Hoxb1*, *Hand1*, *Mesp2*, *Pax3, Mab21l2, Mef2c* and *Epcam* in gastruloids at day 6. Scale bars represent expression levels. Numbers represent Leiden clusters.

day 11

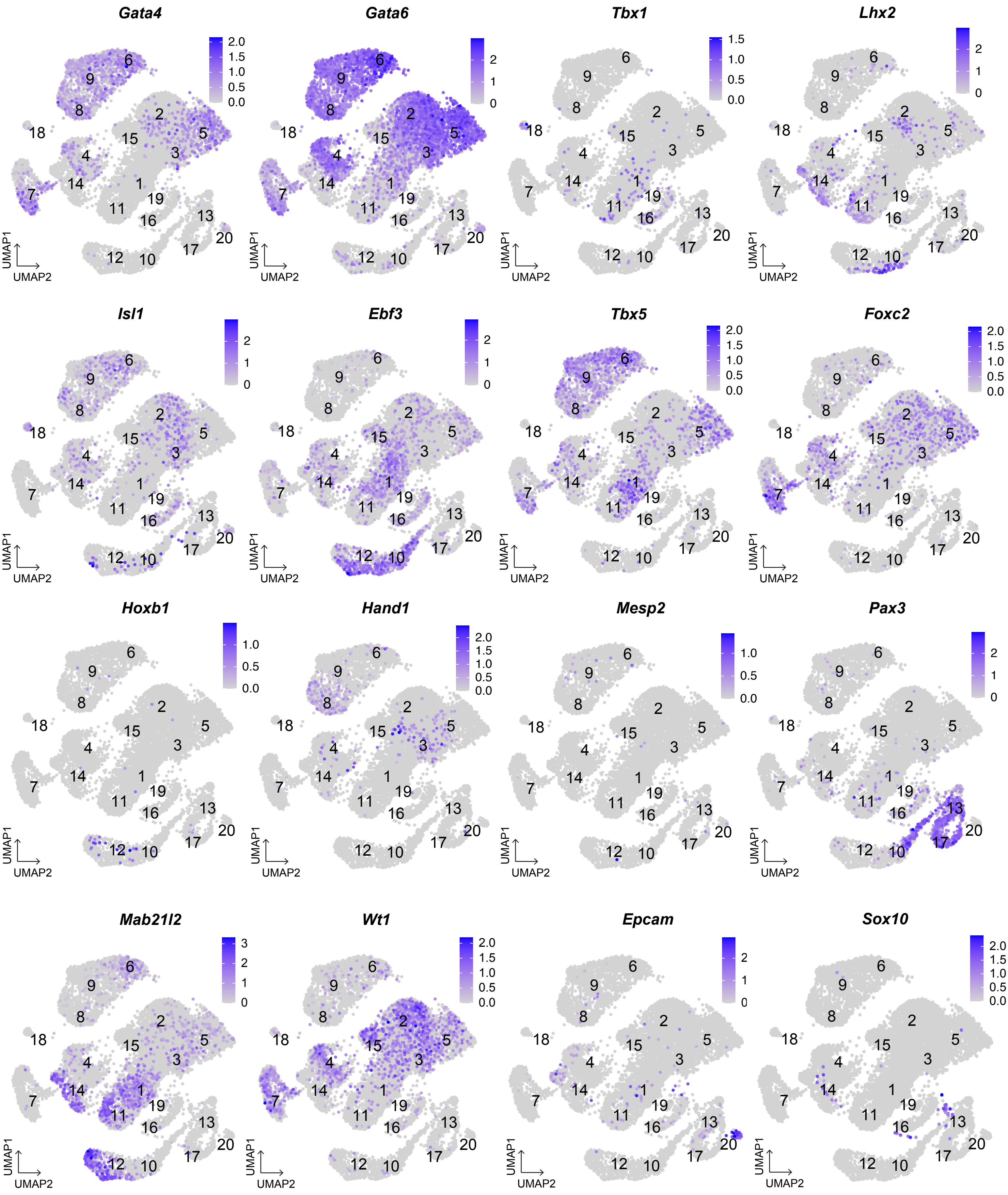

Supplementary Figure 12. Expression of key markers in single-cell RNAseq of gastruloids at day 11. Feature Plots showing expression of *Gata4*, *Gata6*, *Tbx1*, *Lhx2*, *Isl1*, *Ebf3*, *Tbx5*, *Foxc2*, *Hoxb1*, *Hand1*, *Mesp2*, *Pax3*, *Mab21l2*, *Wt1*, *Epcam* and *Sox10* in gastruloids at day 11. Scale bars represent expression levels. Numbers represent Leiden clusters.

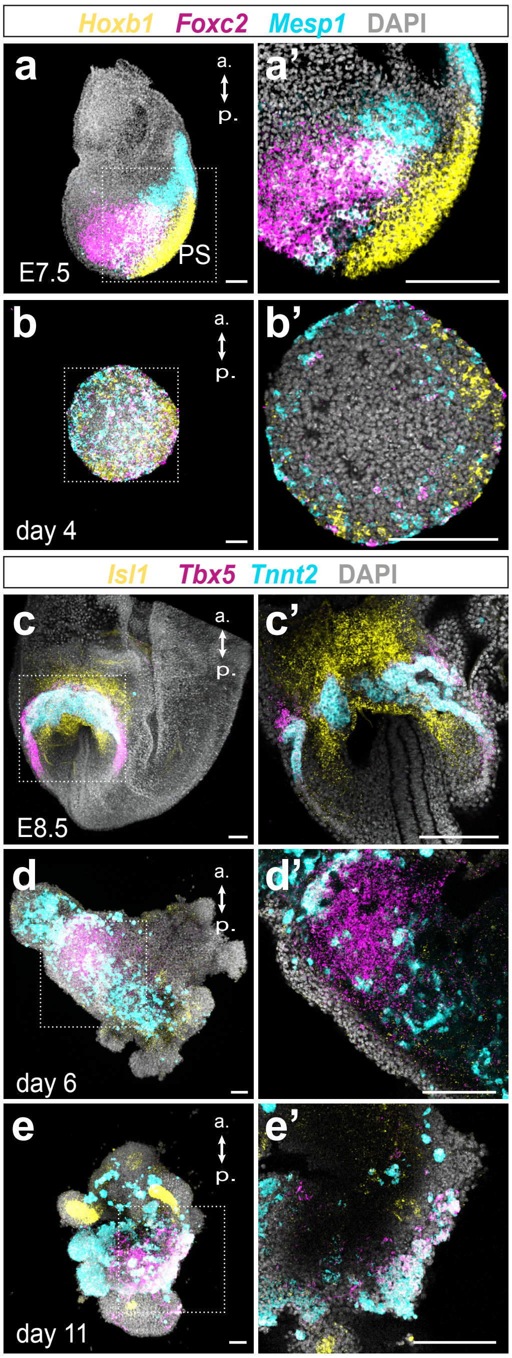

Supplementary Figure 13. Expression of markers of FHF, aSHF and pSHF markers in embryos and gastruloids.

a-b. Representative maximum intensity projection images of a mouse embryo at E7.5 (early-late bud stage) (**a**) and a gastruloid on early day 4 (**b**) after RNAscope experiment with *Hoxb1* (pSHF, yellow), *Foxc2* (aSHF, purple) and *Mesp1* (cyan) probes. Optical sections of the area highlighted by the dotted square is shown in **a'** and **b'**. n=2 embryos and >5 gastruloids in 2 independent experiments. **c-e.** Representative maximum intensity projection images of a mouse embryo at E8.5 (**c**) and gastruloids on day 6 (**d**) and on day 11 (**e**) after RNAscope experiment with *Isl1* (SHF, yellow), *Tbx5* (FHF or pSHF, purple) and *Tnnt2* (cyan) probes. Optical sections of the area highlighted by the dotted square is shown in **c'**, **d'** and **e'**. n=2 embryos and >5 gastruloids in at least 2 independent experiments. Scales: 100µm. PS, primitive streak, a, anterior, p, posterior.

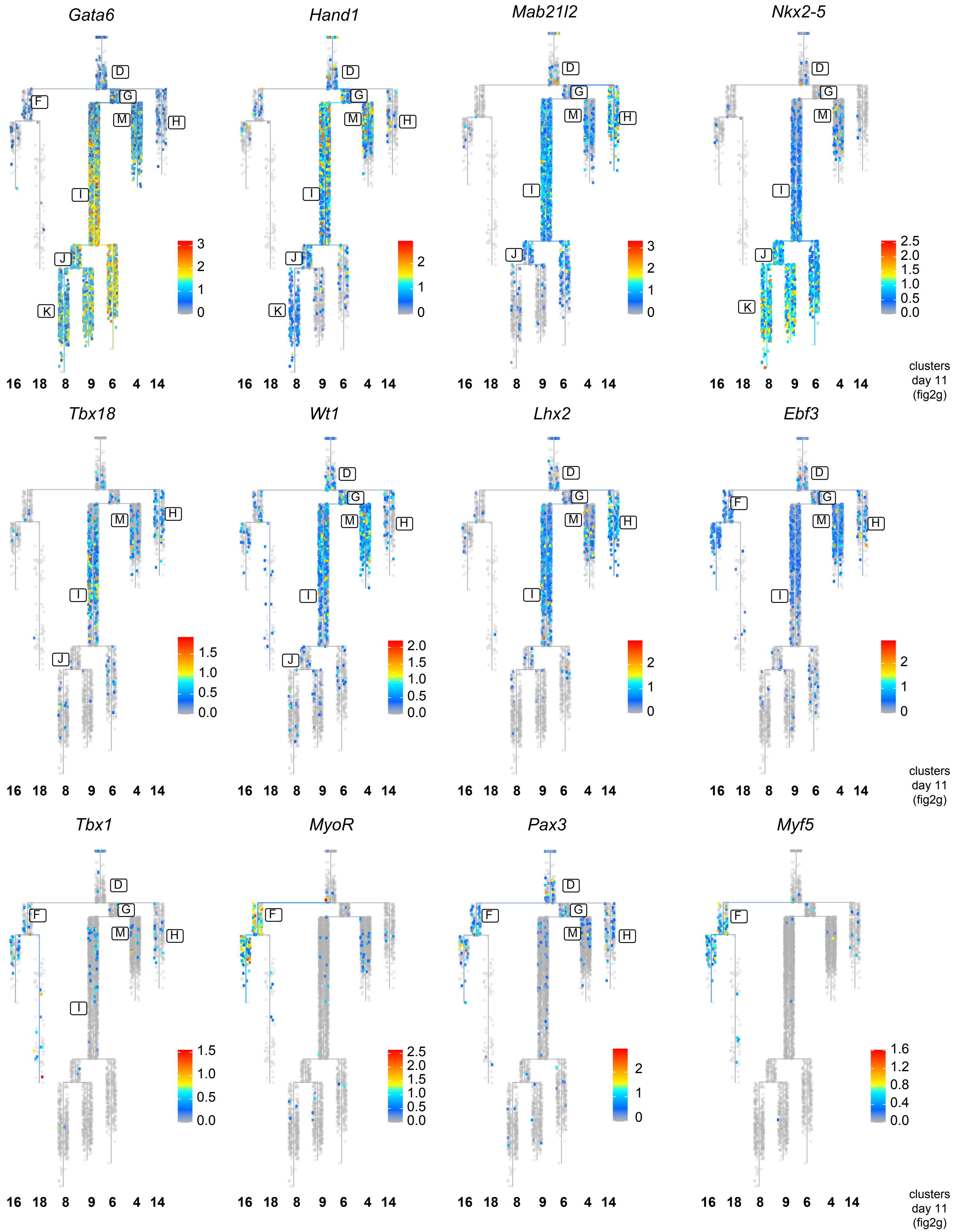

Supplementary Figure 14. Expression of key markers across the URD trajectory.
Expression of *Gata6*, *Hand1*, *Mab21l2*, *Nkx2-5*, *Tbx18*, *Wt1*, *Lhx2*, *Ebf3*, *Tbx1*, *MyoR*, *Myf5* and *Pax3* across URD trajectory. Scale bars represent expression levels.

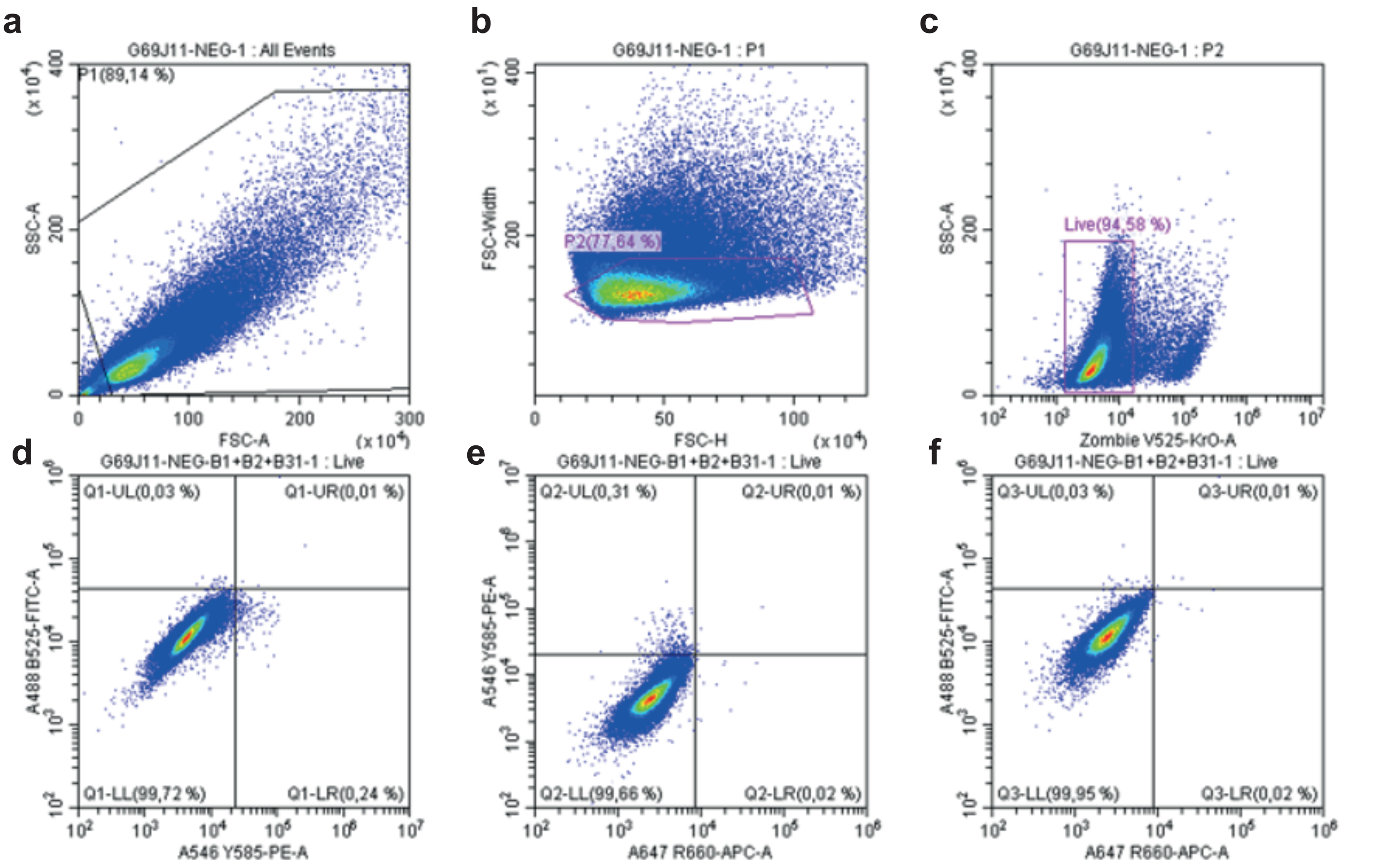

Supplementary Figure 15. Gating strategy for HCR flow cytometry.

a. FSC-A/SSC-A (FSC-A, forward scatter area; SSC-A, side scatter area) plots show gated cells. **b.** FSC-Width/FSC-H (FSC-height) plot show gated singlet cells. **c.** The viable cells were gated in SSC-A/Zombie plot. **e-f.** Plots with negative cells to show gating strategy for FITC(A488)/PE (A546) (**d**), PE (A546)/APC (A647) (**e**) and FITC (A488)/ APC (A647) (**f**) positive signal. The cell population density is color coded (red,high density; blue, low density).
