# Supplementary material for: Gastruloids are competent to specify both cardiac and skeletal muscle lineages
Source: Nat Commun. 2024 Nov 23;15:10172. doi: 10.1038/s41467-024-54466-w (PMC11585638; doi:10.1038/s41467-024-54466-w)
Supplement: Supplementary file 3 — Description of Additional Supplementary Files [file 41467_2024_54466_MOESM3_ESM.pdf]

Description of Additional Supplementary Files

File Name: Supplementary Data 1
Description: Differential gene expression analysis at day 4

File Name: Supplementary Data 2
Description: Differential gene expression analysis (between the myoblasts clusters of day 11)

File Name: Supplementary Movie 1
Description: **Beating gastruloid (Zx1 mESC line) at day 11.** Movie of a beating gastruloids with the Zx1 mESC line at day 11. Scale bar: 100µm.

File Name: Supplementary Movie 2
Description: **Beating gastruloid (Mef2c-Cre; Rosa-tdTomato mESC line) at day 11**. Movie of a beating Mef2c-Cre; Rosa-tdTomato gastruloids at day 11. Scale bar: 100µm.
