# Supplementary material for: Gastruloids are competent to specify both cardiac and skeletal muscle lineages
Source: Nat Commun. 2024 Nov 23;15:10172. doi: 10.1038/s41467-024-54466-w (PMC11585638; doi:10.1038/s41467-024-54466-w)
Supplement: Supplementary file 8 — Reporting Summary [file 41467_2024_54466_MOESM8_ESM.pdf]

nature portfolio

Corresponding author(s): Lescroart Fabienne and Zaffran stéphane

Last updated by author(s): Sep 20, 2024

Reporting Summary

Nature Portfolio wishes to improve the reproducibility of the work that we publish. This form provides structure for consistency and transparency in reporting. For further information on Nature Portfolio policies, see our Editorial Policies and the Editorial Policy Checklist.

Statistics

For all statistical analyses, confirm that the following items are present in the figure legend, table legend, main text, or Methods section.

n/a	Confirmed	
☐	☒	The exact sample size (*n*) for each experimental group/condition, given as a discrete number and unit of measurement
☐	☒	A statement on whether measurements were taken from distinct samples or whether the same sample was measured repeatedly
☐	☒	The statistical test(s) used AND whether they are one- or two-sided<br>*Only common tests should be described solely by name; describe more complex techniques in the Methods section.*
☒	☐	A description of all covariates tested
☒	☐	A description of any assumptions or corrections, such as tests of normality and adjustment for multiple comparisons
☐	☒	A full description of the statistical parameters including central tendency (e.g. means) or other basic estimates (e.g. regression coefficient) AND variation (e.g. standard deviation) or associated estimates of uncertainty (e.g. confidence intervals)
☐	☒	For null hypothesis testing, the test statistic (e.g. *F*, *t*, *r*) with confidence intervals, effect sizes, degrees of freedom and *P* value noted<br>*Give P values as exact values whenever suitable.*
☒	☐	For Bayesian analysis, information on the choice of priors and Markov chain Monte Carlo settings
☐	☒	For hierarchical and complex designs, identification of the appropriate level for tests and full reporting of outcomes
☒	☐	Estimates of effect sizes (e.g. Cohen's *d*, Pearson's *r*), indicating how they were calculated

Our web collection on statistics for biologists contains articles on many of the points above.

Software and code

Policy information about availability of computer code

Data collection	Confocal imaging:<br>All sections, wholemount gastruloids or embryos were imaged using a Zeiss LSM800 confocal microscope with the lattest version of the Zen software.<br><br>Flow cytometry (Mef2c-Cre/tdTomato):<br>A BD FACS Aria II cytometer was used.<br><br>HCR RNA flowcytometry:<br>Data were collected on a a Beckman Coulter Cytoflex LX.<br><br>qPCR data collection was performed on the QuantStudio 5 Dx Real-Time PCR System (ThermoFisher)<br><br>Next Generation sequencing: single cell isolation was performed on the Chromium controlller (10X genomics) . Sequencing of the DNA libraries was performed using an Illumina NextSeq 500 at the MMG Sequencing platform.
Data analysis	Image analysis: Images were analyzed using Zen v3.9 and v3.10 software from Zeiss, ImarisViewer v10.1.0.<br><br>All packages used for scRNAseq analysis are available online and the versions and programs are described in the Materials and Methods section. For more details , the analysis was implemented in R v4.1.3 and Python v3.9.10. We have used the following packages/tools: Seurat

v4.1.1, DoubletFinder v2.0.3, uwot v0.1.14, CellRank v1.5.1, Scanpy v1.9.1, URD v1.1.1.

For manuscripts utilizing custom algorithms or software that are central to the research but not yet described in published literature, software must be made available to editors and reviewers. We strongly encourage code deposition in a community repository (e.g. GitHub). See the Nature Portfolio guidelines for submitting code & software for further information.

Data

Policy information about availability of data

All manuscripts must include a data availability statement. This statement should provide the following information, where applicable:

- Accession codes, unique identifiers, or web links for publicly available datasets
- A description of any restrictions on data availability
- For clinical datasets or third party data, please ensure that the statement adheres to our policy

The scRNA-seq data generated in this study have been deposited in the Gene Expression Omnibus (GEO) database under accession code GSE232773 https://www.ncbi.nlm.nih.gov/geo/query/acc.cgi. The processed single-cell data are available at https://github.com/BAUDOTlab/gastruloid_timeserie_scRNA-seq. The qPCR and flow cytometry generated in this study are provided in the Supplementary Information/Source Data file.

Research involving human participants, their data, or biological material

Policy information about studies with human participants or human data. See also policy information about sex, gender (identity/presentation), and sexual orientation and race, ethnicity and racism.

Reporting on sex and gender	N/A
Reporting on race, ethnicity, or other socially relevant groupings	N/A
Population characteristics	N/A
Recruitment	N/A
Ethics oversight	N/A

Note that full information on the approval of the study protocol must also be provided in the manuscript.

Field-specific reporting

Please select the one below that is the best fit for your research. If you are not sure, read the appropriate sections before making your selection.

- [x] Life sciences
- [ ] Behavioural & social sciences
- [ ] Ecological, evolutionary & environmental sciences

For a reference copy of the document with all sections, see nature.com/documents/nr-reporting-summary-flat.pdf

Life sciences study design

All studies must disclose on these points even when the disclosure is negative.

Sample size	For scRNAseq, qPCR experiments, sample sizes were estimated based upon requirements for optimal cell numbers. In situ hybridization, immunofluorescence were done in at least n=3 embryos/gastruloid. Samples size for each experiments is indicated in the figures or corresponding legends. No statistical methods were used to determine sample size, n equal or superior to 3 samples is the standard used by convention in the research field.
Data exclusions	All data were included for the analysis.
Replication	All the experiments (except scRNAseq) were performed in at least 2 biologically independent replicates. All attempts at replication of the results were successful.
Randomization	Since embryos were not genotyped for the presence of the Y chromozome, sex-specific differences were minimed by including randomized numbers of male and female embryos. Embryos and gastruloids were randomly allocated in the different experiments.
Blinding	All analyses were unbiased. scRNAseq uses software that provides unbiased clustering and peaks.

Reporting for specific materials, systems and methods

We require information from authors about some types of materials, experimental systems and methods used in many studies. Here, indicate whether each material, system or method listed is relevant to your study. If you are not sure if a list item applies to your research, read the appropriate section before selecting a response.

Materials & experimental systems

n/a	Involved in the study	
☐	☒	Antibodies
☐	☒	Eukaryotic cell lines
☒	☐	Palaeontology and archaeology
☐	☒	Animals and other organisms
☒	☐	Clinical data
☒	☐	Dual use research of concern
☒	☐	Plants

Methods

n/a	Involved in the study	
☒	☐	ChIP-seq
☐	☒	Flow cytometry
☒	☐	MRI-based neuroimaging

Antibodies

Antibodies used

The following primary antibodies were used:
- anti-cTnT (1:100-400, Invitrogen MA5-12960 clone 13-11)
- goat anti-VEGFR2 (1:100, R&D AF644)
- rat anti-E-cadherin (1:100, Takara M108 clone eccd-2)
- mouse anti-MyoG (1:10, DSHB, F5D-S)

The following secondary antibodies were used:
- donkey anti-mouse-IgG Alexa555 (1:400, Invitrogen A-31570)
- donkey anti-goat-IgG Alexa647 (1:400, Invitrogen A-21447)
- donkey anti-rat-IgG Alexa488 (1:400, Invitrogen A-21208)
- goat anti-mouse-IgG Alexa647 (1:400, Invitrogen A-12235)
- anti-mouse IgG PE-Cy7 (1:200, Biolegend, 406613)

Validation

- anti-cTnT (1:100-400, Invitrogen MA5-12960): Lin et al. 2022 Nature Cell Biology and https://www.thermofisher.com/antibody/product/Cardiac-Troponin-T-Antibody-clone-13-11-Monoclonal/MA5-12960
- goat anti-VEGFR2 (1:100, R&D AF644): https://www.rndsystems.com/products/mouse-vegfr2-kdr-flk-1-antibody_af644?gad_source=1&gclid=Cj0KCQjwo8S3BhDeARIsAFRmkOOAf0fwYxyJ_EPf7-XMkVGj8FcV6bTmq4hCyUlNCYSJv1FTIgdoWIEaAubBEALw_wcB&gclsrc=aw.ds
- rat anti-E-cadherin (1:100, Takara M108): Hashmi et al. 2022 eLife
- mouse anti-MyoG (1:10, DSHB, F5D-S): Nandkishore et al. 2018 Development

Eukaryotic cell lines

Policy information about cell lines and Sex and Gender in Research

Cell line source(s)	R1/E #1036 male mouse embryonic ES cells were purchased from ECACC (cat. Number 07072001). Zx1 male mouse ESCs were obtained from M. Kyba (Iacovino et al. 2011 Stem Cells). Mef2c-cre Rosa tdTomato male cells were derived in the lab from E3.5 bastula collected from mouse (Verzi et al. 2005 Developmental Biology). Males ESC lines were used to minimize cell line to cell line variations.
Authentication	The mESC lines for this study were not authenticated.
Mycoplasma contamination	All cell lines have been tested negative for mycoplasma contamination.
Commonly misidentified lines (See ICLAC register)	No commonly misidentified lines were used in this study.

Animals and other research organisms

Policy information about studies involving animals; ARRIVE guidelines recommended for reporting animal research, and Sex and Gender in Research

Laboratory animals	Mef2c–AHF-Cre and Rosa-tdTomato (Gt(ROSA)26Sortm9(CAG-tdTomato)Hze) mice were previously described (Verzi et al. 2005 Developmental Biology). We used CD1/Swiss mice (Charles River) as wildtype animals. Adult breeding mice in our colony range 2-8 months age. We did not genotype for sex of the embryos. Mice were housed at a temperature betweeen 20 to 22°C with a level of humidity between 40 to 60%. Each cage was provided with food, water and two types of nesting materials. Mice colonies were maintained in a certified animal facility with 7h to 19h light cycle.
Wild animals	No wild animals were involved in this study.
Reporting on sex	We did not genotype for sex of the embryos because we were examinating stages prior to sexual development.
Field-collected samples	No field collected samples were used in this study.
Ethics oversight	Mouse colonies were maintained in certified animal facilities (agreement #C 13 013 08) in accordance with European guidelines. The

experiments were approved by the local ethical committee (Aix-Marseille Univ CEBEA CE14 directed by Erica Lopez) and the study is compliant with all relevant ethical regulations regarding animal research (Ministère de l'Education Nationale, de l'Enseignement Supérieur et de la Recherche; Authorization N 32-08102012).

Note that full information on the approval of the study protocol must also be provided in the manuscript.

Plants

Seed stocks	N/A
Novel plant genotypes	N/A
Authentication	N/A

Flow Cytometry

Plots

Confirm that:

- [x] The axis labels state the marker and fluorochrome used (e.g. CD4-FITC).
- [x] The axis scales are clearly visible. Include numbers along axes only for bottom left plot of group (a 'group' is an analysis of identical markers).
- [x] All plots are contour plots with outliers or pseudocolor plots.
- [x] A numerical value for number of cells or percentage (with statistics) is provided.

Methodology

Sample preparation

For Mef2c-Cre/tdTomato flow analysis:
Gastruloids collected on day 11 were dissociated by incubation 10 min at 37°c in 0,5% Trypsin-EDTA. Cells were washed 2 times with D-MEM + 10% FBS and filtrated through a 40µ cell strainer (Falcon 352340). After a PBS wash, 1 million cells were incubated for 20 min at room temperature with Zombie viability dye (Biolegend ref 77143). Then cells were incubated in PBS + 10% FBS to block Fc receptors, followed by a permeabilization/fixation with Cytofix/Cytoperm BD kit (ref 554714). After washing, mouse anti-cTnT antibody (Invitrogen MA5-12960) was incubated at a 1/400 dilution in BD wash buffer during 20 min at room temperature. Then cells were again washed and followed by an incubation with anti-mouse IgG coupled to PE-Cy7 (Biolegend ref 406613) at a 1/200 dilution in BD wash buffer during 20 min at room temperature. After three washes, cells were resuspended in PBS + 1% BSA (Sigma ref A8412-100ML), filtered on a 40µ cell strainer.

For HCR flow cytometry:
Gastruloids collected on day 11 were dissociated by incubation 10 min at 37°c in 0,5% Trypsin-EDTA. Cells were washed 2 times with PBS + 10% FBS and filtrated through a 40µ cell. After PBS wash, 1 million cells were incubated for 20 min at room temperature with Zombie viability dye. Cells were then fixed by incubation with 4% PFA (EMS15714) during 1h at room temperature. HCR multiplexed RNA detection was realized according to Molecular Instruments protocols. Cells were washed in PBST: PBS + 0,1% Tween 20, resuspended in 70% Ethanol and kept overnight at 4°c. The day after, cells were washed twice in PBST and incubated at 37°c in hybridization buffer. Probes specific for the different genes were then added at 16nM concentration and incubated overnight at 37°c. Cells were then washed 5 times with wash buffer and 2 times with 5x SSC (Gibco ref 15557-044) + 0,1% Tween 20. Then incubation of the cells with amplification buffer was done during 30 min at room temperature. Amplification hairpin h1 and h2 were prepared separately by heating at 95°C for 90 sec and cooling to room temperature for 30 min. Then hairpins were pooled at 60 nM and incubated with cells overnight at room temperature. After 6 washes with 5x SSC + 0,1% Tween 20. Samples were filtered on Flowmi 70µm (Bel-Art H136800070).

Instrument

For Mef2c-Cre/tdTomato flow analysis: BD FACS Aria II cytometer.
For HCR flow cytometry: Beckman Coulter Cytoflex LX.

Software

For Mef2c-Cre/tdTomato flow analysis: FACS Diva v9.0.1 (BD Biosciences)
For HCR flow cytometry: CytExpert v2.5 (Beckman-Coulter)

Cell population abundance

A minimum of 100 000 cells per samples were analyzed.

Gating strategy

We excluded debris according to size (FSC<20, SSC<20). Live cells were based on Zombie Aqua Fixable Viability Kit negative (<1.10^4). 7x10^3 and above was set as fluorescent signal for PE-Cy7 anti-TnT, 3x10^3 and above was set as fluorescent signal positive for tdTomato as shown in Fig.4k. 4x10^4 and above was set as fluorescent signal positive for Alexa488 Myl7 and 1.5 X 10^4 and above was set as fluorescent signal positive for Alexa546 Myl2 as shown in Fig. 4i and supplementary Fig. 15. 1^10^4 and above was set as fluoresecent positive signal for Alexa546 Myh3 and 8x10^3 and above was set as

fluorescent signal positive for Alexa647 Myog as shown in Fig. 5h and Supplementary Fig. 15.

☒ Tick this box to confirm that a figure exemplifying the gating strategy is provided in the Supplementary Information.
